# Developing halal consumer behavior and tourism studies: Recommendations for Indonesia and Spain

**DOI:** 10.3389/fpsyg.2022.863130

**Published:** 2022-08-05

**Authors:** Citra Kusuma Dewi, Mahir Pradana, Rubén Huertas-García, Nurafni Rubiyanti, Syarifuddin Syarifuddin

**Affiliations:** ^1^Department of Business Administration, Telkom University, Bandung, Indonesia; ^2^Department of Business, University of Barcelona, Barcelona, Spain

**Keywords:** halal, consumer behavior, halal accessibility, Islamic, consumer

## Introduction

In recent years, there have been several studies conducted on intentions of Muslim consumers to measure the right attitude in buying halal food so that business development of halal food products can be augmented (Wilson, 2014). Moreover, as revealed by Sandikçi (2011), acceptance of halal products is also increasingly widespread among non-Muslims. Furthermore, researchers on business and psychology have been busy in examining this phenomenon either in Muslim or non-Muslim countries.

It is essential for Islamic believers that the category of the products they consume is halal or haram (Madiawati and Pradana, 2016). “Haram” is unlawful according to Islamic holy bible (Al Qur'an) (Alserhan, 2010). In reality, some groups of Muslim consumers in certain countries still do not have access to sufficient information about halal certification or halal logo (Pradana et al., 2021). On the other hand, there is a lack of academic research regarding the effect or relationship between halal concepts and consumer purchase intention, in this case, consumers' attitude or subjective norms, halal certification and halal food purchase intention (Aziz and Chok, 2013).

At the world population level, Islam is a religion with a fairly large number of followers, reaching 23 percent (Garg and Joshi, 2018). Around 52 percent of the world's Muslims are still in the category of young consumers, making Muslims a significant and large potential market (Wilson, 2014). Several prominent publications related to this topic are Bonne and Verbeke (2008), Tieman et al. (2013), and many more.

In this article, we discuss the literature observing the behavior of the Indonesian and Spanish Muslim communities in purchasing halal products. Indonesia is an interesting location to conduct such study, mainly for the reason that it can be a representation of the world's Muslim community, while Spain is taken as a comparison because of the author's previous studies on related matter (Pradana et al., 2019, 2020, 2021).

## Halal consumer behavior literature in Indonesia

In this case, the potential main study object is Indonesia. It is one of the largest contributors to the world's Muslim population, reaching over 12.6% in 2019 (Madiawati et al., 2021). The market potential for Muslims will continue to grow, as revealed by the Pew Research Institute, which predicts that by 2070 Muslims will become the largest religious group in the world (Madiawati and Pradana, 2016).

Studies have argued on how halal food consumers in different countries have different perspectives and levels of trust on the concept of halal certification (Rios et al., 2014; Meixner et al., 2018). Indonesia, as the largest Muslim country, already has a sufficient number of publications related to this matter (Prabowo et al., 2015; Adinugraha et al., 2019). Other prominent publications related to this topic can be seen in Table 1.

**Table 1:** Some Indonesian publications on halal topics.

**Number**	**References**	**Titles**	**Findings**	**Journal**	**Year**
1	Jaelani, 2017	Halal tourism industry in Indonesia: potential and prospects	Halal tourism industry can not be separated from the religious practices of the majority of Muslims in Indonesia, but also economically contribute to the local community, and tourist sites	International Review of Management and Marketing	2017
2	Sukesti and Budiman, 2014	The influence halal label and personal religiousity on purchase decision on food products in Indonesia	The results showed halal label and personal religiousity significantly influence the purchase decision	International Journal of Business, Economics and Law	2014
3	Hudaefi and Jaswir, 2019	Halal governance in Indonesia: theory, current practices, and related issues	This paper offers explanation of lines of defense in halal governance, and that of the current practices in Indonesia	Journal of Islamic Monetary Economics and Finance	2019
4	Vanany et al., 2019	Determinants of halal-food consumption in Indonesia.	Attitudes, religious self-identity and moral obligations were significant predictors of intention to consume halal food.	Journal of Islamic Marketing	2019

Source: authors' own elaboration.

If we take a deeper look at the halal literature on Indonesia, it has not focused on halal tourism development. We might argue that Indonesia might not regard it as an essential topic since the majority of Indonesian population is Muslim. However, Indonesia is a potential destination for tourists from the Middle East who are mostly Muslims (KataData, 2017). Therefore, we suggest that more studies on halal tourism should be conducted in Indonesia, next to studies on halal consumer behavior.

## Halal literature in Spain

Spain has a long Islamic history, dating back to the Moors' reign in 800 A.D (Mesa, 2012). Islamic consumption of halal foods in Spain has increased, proven by the facts that in Spain 62 percent of lamb and 56 percent of beef come from animals slaughtered according to Islamic law (Vargas-Sánchez and Moral-Moral, 2019).

Spain is currently expanding exhibitions for halal food and will increase the number of participating companies specializing in this type of food (Pradana et al., 2019). It will also host the third edition of the Halal Congress, which will include international experts and several cooking events (Bottoni, 2021). There is also a greater demand from non-Muslim consumers for products that are organic and healthy and that have a halal certification as a guarantee of quality (Abbasian, 2021). However, academic approaches supporting the growing halal market in Spain are limited. Vargas-Sánchez and Moral-Moral (2019) and Pradana et al. (2020) are among the publications that focused on this region. The limited number of publications can be seen in Table 2.

**Table 2:** Some Spanish publications on halal topics.

**Number**	**References**	**Titles**	**Findings**	**Journal**	**Year**
1	Vargas-Sánchez and Moral-Moral, 2019	Halal tourism: state of the art.	Halal tourism is a field of study still in a very early stage; however, on a practical level, the number of halal products and services is starting to increase worldwide.	Tourism Review	2019
2	Vargas-Sánchez and Moral-Moral, 2019	Halal tourism: literature review and experts' view	Halal tourism is a field of study that is still in a very early stage. It can be understood as the offer of tourist services designed to meet the needs of Muslim tourists in accordance with their religious obligations.	Journal of Islamic Marketing	2019
3	Pradana et al., 2020	Spanish Muslims' halal food purchase intention	Product awareness does not have an effect on purchase intention while the mediating effect of consumers' attitude toward halal label and moderating effect of religious involvement.	International Food and Agribusiness Management Review	2020
4	Pradana et al., 2021	Muslim tourists' purchase intention of halal food in Spain	Our result shows that both the halal credence and the need for cognition have no direct effects on halal purchase intention. However, halal consumers' attitude acts as significant mediators in the indirect effects of both halal credence and the need for cognition on halal purchase intention.	Current Issues in Tourism	2021

Source: authors' own elaboration.

Although the numbers are few, three main publications regarding halal topics in Spain focus on tourism. The academic discussion has become popular in the last few years, mainly because Spain had welcomed over five million Muslim tourists until 2019, visiting ancient Islamic sites in Cordoba and Granada (Vargas-Sánchez and Moral-Moral, 2019; Walker, 2019). However, halal tourism in Spain remains stagnant although the country has been reaping Muslim visitors (Pradana et al., 2021).

Therefore, it is our offered future research recommendation that halal consumer behavior and halal tourism literature in Spain should be expanded. Comparison with the Indonesian case and collaboration between Indonesian and Spanish researchers can be beneficial in boosting the number of Spanish academic publications related to this matter. Both Indonesia and Spain need to explore halal tourism more, while Indonesian researchers can assist their Spanish counterparts in developing halal consumer behavior literature.

## Author contributions

CD, RH-G, NR, MP, and SS discussed, wrote, and finalized the manuscript together. All authors contributed to the article and approved the submitted version.

## Conflict of interest

The authors declare that the research was conducted in the absence of any commercial or financial relationships that could be construed as a potential conflict of interest.

## Publisher's note

All claims expressed in this article are solely those of the authors and do not necessarily represent those of their affiliated organizations, or those of the publisher, the editors and the reviewers. Any product that may be evaluated in this article, or claim that may be made by its manufacturer, is not guaranteed or endorsed by the publisher.
